# A swarm of autonomous miniature underwater robot drifters for exploring submesoscale ocean dynamics

**DOI:** 10.1038/ncomms14189

**Published:** 2017-01-24

**Authors:** Jules S. Jaffe, Peter J. S. Franks, Paul L. D. Roberts, Diba Mirza, Curt Schurgers, Ryan Kastner, Adrien Boch

**Affiliations:** 1 Scripps Institution of Oceanography, University of California San Diego, La Jolla, California 92093, USA; 2 Computer Science and Engineering, University of California San Diego, La Jolla, California 92093, USA; 3 Qualcomm Institute, University of California San Diego, La Jolla, California 92093, USA

## Abstract

Measuring the ever-changing 3-dimensional (3D) motions of the ocean requires simultaneous sampling at multiple locations. In particular, sampling the complex, nonlinear dynamics associated with submesoscales (<1–10 km) requires new technologies and approaches. Here we introduce the Mini-Autonomous Underwater Explorer (M-AUE), deployed as a swarm of 16 independent vehicles whose 3D trajectories are measured near-continuously, underwater. As the vehicles drift with the ambient flow or execute preprogrammed vertical behaviours, the simultaneous measurements at multiple, known locations resolve the details of the flow within the swarm. We describe the design, construction, control and underwater navigation of the M-AUE. A field programme in the coastal ocean using a swarm of these robots programmed with a depth-holding behaviour provides a unique test of a physical–biological interaction leading to plankton patch formation in internal waves. The performance of the M-AUE vehicles illustrates their novel capability for measuring submesoscale dynamics.

## Abstract (web summary)

Observing dynamics associated with oceanic submesoscales requires simultaneous sampling at multiple locations. Here, the authors show that a swarm of 16 Mini-Autonomous Underwater Explorers (M-AUEs), whose 3D trajectories are measured near-continuously, can resolve features of the flow at these scales.

Owing to the ocean's vast size, inaccessibility, intense pressure, corrosive environment and optical opacity, developing technologies to study the oceans has always been difficult. The introduction of *in situ* electronic sensors such as CTDs (conductivity–temperature–depth), acoustic systems and chlorophyll fluorometers revolutionized oceanography in the late twentieth century by reducing the need to acquire and recover water samples for shipboard analysis and by offering orders of magnitude better spatial and temporal resolution of important oceanographic properties than previously available through bottle or net sampling^1^.

Some of the least understood physical dynamics in the ocean occur between scales of a metre and a few kilometres. These include submesoscale processes, internal waves and the transition from internal waves to turbulence^2^,^3^. Many of these physical flows are intensified in the upper layers of the ocean; their scales of variability make them particularly relevant to fundamental biological dynamics, from driving patchiness in primary production to subduction of fixed organic carbon to transport of meroplanktonic larvae^4^. These physical and biological dynamics occur at spatial and temporal scales that are difficult or impossible to resolve in three dimensions (3D) with traditional moored, profiling, towed or remote-sensing instruments.

Lagrangian (current-following) vehicles are ideal for studying ocean dynamics at submesoscales, as they move with the water, allowing reconstruction of the 3D movements of water parcels down to the scales of the vehicles themselves^5^,^6^. Furthermore, swarms of drifting vehicles provide a significant enhancement over single vehicles by allowing the calculation of spatial and temporal gradients of properties such as velocity, vorticity or temperature. Significantly, large numbers of vehicles in a swarm allow quantification of changes in their concentration—their patchiness—and its relation to the underlying flow.

The deployment of large numbers of subsurface drifting vehicles was first envisioned by Stommel^7^ and realized by Swallow^8^,^9^, who deployed up to 22 floats simultaneously^10^. Subsurface positions of these floats were obtained acoustically once per day, with an accuracy of about ±0.5 km. The ongoing Argo program (2016; http://www.argo.ucsd.edu) currently has almost 4000 floats throughout the global ocean, providing unique data on global circulation patterns and water properties. Satellite fixes are obtained about every 10 days when the floats come to the surface, with several kilometre accuracy owing to lateral displacements during the ascents, in addition to the uncertainty in the satellite positioning. Recently, a swarm of 18 EM-APEX profiling drifters^11^ was deployed in a ∼2 km diameter patch during the LatMix experiment^12^. These floats profiled continuously between the surface and 150 m, obtaining fixes when at the surface—every 50 min or so. However, despite the significant advances in using drifters to sample the large-scale dynamics of the ocean, there has been no deployment of a swarm of such autonomous subsurface vehicles, continuously tracked underwater, to study physical and biological dynamics in the upper ocean on the biologically relevant scales of metres to kilometres and minutes to days.

One particular process amenable to measurement by a swarm of subsurface drifters is plankton subsurface patch formation in high-frequency internal waves. Intense phytoplankton patches often appear as kilometer-long, ∼100-m wide bands parallel to shore, presumably associated with subsurface high-frequency internal waves^13^,^14^,^15^. The plankton are hypothesized to depth hold, swimming against the internal-wave-induced flows, thereby accumulating in regions where the ambient horizontal velocities converge^13^. This interaction of weakly swimming plankton with the convergence of horizontal currents—the horizontal strain—is thought to be a primary mechanism forming plankton patches in the ocean at these scales^16^,^17^,^18^. The plankton need no knowledge of each other's location or concentration; if they all have the same swimming behaviour, they will tend to accumulate in horizontal convergences and disperse in divergences.

High-frequency internal waves are a dominant source of horizontal strain in coastal waters. As these waves propagate within the ocean, they create a convergence zone behind the crest of the wave. Theoretical models predict that depth-holding plankton will accumulate in the wave troughs^13^,^14^,^15^, where the horizontal strain changes from convergent to divergent.

Here we introduce the Mini-Autonomous Underwater Explorer (M-AUE), which we deploy as a swarm of 16 independent vehicles that can move with the horizontal water flows, while at the same time having preprogrammed vertical swimming behaviours and near-continuous, 3D, underwater tracking. The distributed array formed by the vehicles in the swarm permits measurement of the underlying horizontal flows within the swarm. The M-AUE was designed to be small—smaller than any existing quasi-Lagrangian vehicle we are aware of. Its 1.5 litre displacement volume contains a densely packed set of mechanical and electrical components that allow it to measure horizontal water flows while remaining neutrally buoyant, vertically migrating or holding vertical position. Our vision is to make it feasible for ocean scientists to deploy large swarms of such miniature vehicles to enable new insights into physical–biological dynamics in the ocean. Here we describe how the M-AUE is controlled and how we estimate its underwater location in 3D. We then document how a swarm of these robots, programmed with a simple swimming behaviour, led to the formation of subsurface accumulations in high-frequency internal waves. Measurements of the time-evolving 3D positions of the M-AUEs allowed us to calculate the wave's frequency, wavelength and amplitude, as well as the relationship of the M-AUE concentration (M-AUEs per unit area) to the underlying waveform. As predicted by theory, the M-AUEs accumulated over the wave troughs and dispersed over the wave crests. This is the first time the swimming/internal wave-patch formation hypothesis has been tested underwater.

## Results

### Overall system design

**Figure 1:**
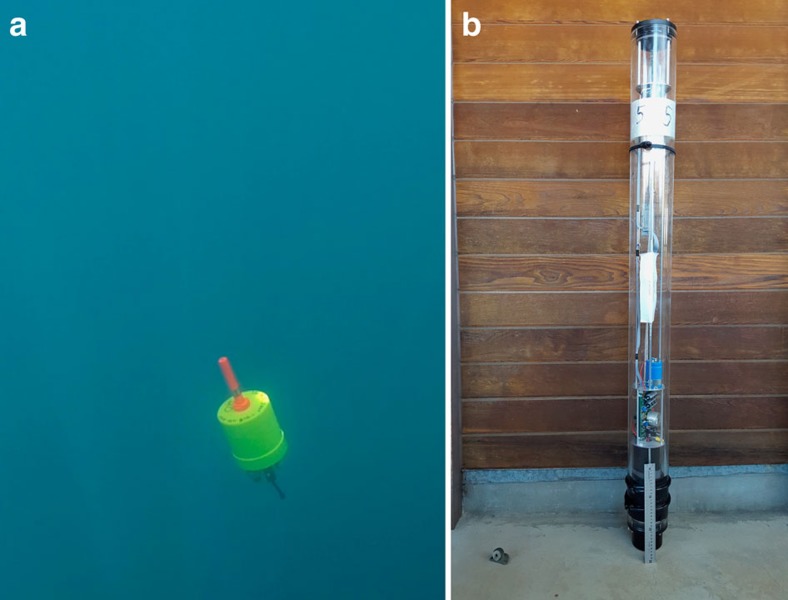
M-AUE and Spar Buoy Pinger. (**a**) The 1.5 litre M-AUE in the ocean at an approximate depth of 3 m. (**b**) The pinger of the GPS-localized spar buoy with a 30 cm ruler at the base for scale.

The M-AUE system is designed to measure time series of 3D ambient currents or to mimic the vertical swimming behaviours of plankton. The overall design goal was to create a portable, easily deployed system that could provide 3D localizations at reasonable update rates in a relevant volume of water. An update rate of 1 min^−1^, 10s of vehicles and an areal coverage of at least 1 km^2^ was determined necessary for sampling internal waves and other submesoscale dynamics. The system consists of two basic components: small, freely drifting subsurface vehicles that can adjust their buoyancy to control depth and whose 3D trajectories can be measured underwater (Fig. 1a), and a set of moored global positioning system (GPS)-localized and synchronized spar buoy surface floats that produce a timed acoustic signal for underwater vehicle navigation (Fig. 1b).

### M-AUE underwater vehicle localization

Typically, underwater vehicles are localized by responding acoustically to the interrogation of a known source, allowing measurement of separation distances via travel times. Unfortunately, generating this acoustic response requires significant energy that would increase the size and cost of the M-AUEs. Instead, acoustic pings generated by the GPS-localized surface floats are passively sensed by the subsurface M-AUEs. Each of the five surface floats transmits their ping in sequence, at precisely controlled times in a ‘round-robin' manner. At the M-AUE, the set of sensed pings is recorded and subsequently combined—via measurement of their send–receive time intervals—with the known positions of the surface float at the time of the ping to estimate the temporally evolving set of M-AUE positions. Vertical positions are obtained via the pressure sensors on the M-AUEs.

The surface float's ping waveform was a broadband chirp with frequency range from 8–15 kHz. The *i*th set of pings was transmitted with an interfloat pulse interval of *Δt*=2 s: the pinger on float (1, 2, 3, 4, 5) transmitted the pulse with start time *T*_i_ seconds at times (*T*_i_, *T*_i_+*Δt*, *T*_i_+2*Δt*, *T*_i_+3*Δt*, *T*_i_+4*Δt*). A GPS-synchronized clock on the surface float was used to ensure microsecond transmit-time accuracy. This sequence is repeated continuously throughout the deployment. The 2-s interpulse interval ensures that the signal received at the M-AUEs can be uniquely assigned to the appropriate surface float.

The M-AUEs recorded the pings using a high-fidelity hydrophone and analogue-to-digital circuits for waveform recording. Postprocessing with a correlation receiver^19^ allowed estimation of the arrival time of the set of surface pings at each M-AUE with no ambiguity in assigning pings to their respective surface float. In addition, we note that it was generally easy to pick out the direct arrival from the matched filtered M-AUE hydrophone output, eliminating the need for more advanced environmental (sound speed) modelling or matched field processing.

Matched filtered acoustic data from each M-AUE were then processed to yield the two-dimensional (2D) locations of the vehicles in latitude and longitude. This was then combined with the vehicle's measured depth to yield tracks of position in depth, latitude and longitude. The tracking is essentially an underwater analogue to GPS localization using a probabilistic framework. Our tracking method constructs a factor-graph representation of the joint probability density function for the lateral locations of the M-AUEs and surface pingers over the duration of the experiment given estimates of distance to the pingers and bounds on the velocity of the M-AUEs. The Maximum Likelihood estimates of the 2D locations of the M-AUEs are computed by running the sum-product algorithm on the factor-graph as described in our previous work^20^,^21^. This algorithm was tested at sea using a GPS-localized hydrophone suspended at a known depth (an M-AUE mimic) and the five surface-float pingers described above^22^. The algorithm showed very low bias in hydrophone locations, with s.e. on the order of a few metres laterally over a 2-km drift track.

### The surface float pinger

The surface float pinger is a 1.5-m long spar buoy with a submerged acoustic transducer (Fig. 1b). The surface floats receive satellite GPS location information that is time-encoded for postmission processing. For the field experiments, the spar buoys were clipped to a simple, easily deployed and recovered mooring made from a 1/4-inch single braid line with 2:1 scope and a 10-kg shackle as an anchor. More details can be found in the Methods section.

### The M-AUE vehicle

**Figure 2:**
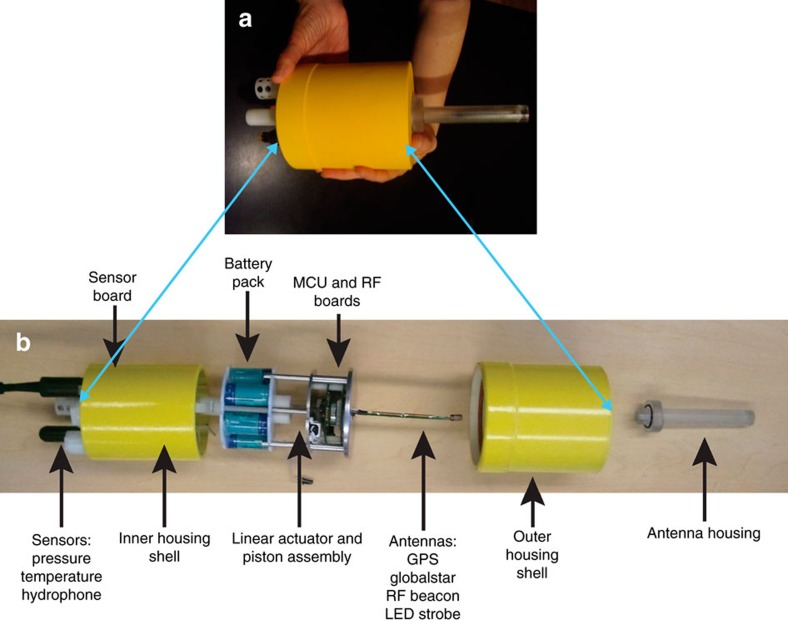
An exploded view of the M-AUE vehicle. (**a**) The M-AUE in closed position. (**b**) The two concentric shells of the M-AUE separated to reveal the internal components.

The M-AUE (Fig. 2) is a self-ballasting, cylindrical, 1.5-litre volume subsurface drifter that can be tracked underwater in 3D. The small size maximizes the surface area to volume ratio, increasing the vehicle's ability to drift with the currents. The vehicle has active buoyancy control to execute vertical movements. To maintain the small size, no propulsion system was incorporated. The M-AUE's electronic and mechanical components were designed to achieve the following mission requirements: it must be hand-deployable from a small boat, acoustically record the surface pings with adequate signal-to-noise ratio to estimate arrival times, execute preprogrammed vertical movements—including depth holding—using buoyancy control, be sufficiently buoyant at the surface to elevate the antenna far enough out of the water for satellite communication, and record depth and temperature. The vehicles need to surface for recovery, then obtain and communicate its GPS position to a retrieving party via satellite, internet or cell phone and have a radio beacon and light-emitting diode strobe to aid in visual recovery. Each vehicle must have sufficient battery energy to perform missions up to several days with a limited number of vertical excursions and have adequate memory to store all vehicle sensor information including the acoustic records, as well as information about the internal state of the vehicle.

The housing of the M-AUE consists of two concentric, syntactic foam cylinders forming inner and outer sleeves that can slide over each other to significantly increase or decrease the vehicle's volume (Fig. 2). This large change in volume allows the vehicle to raise its antenna far enough above the sea surface, when surfaced, so that the satellite links for GPS and the Globalstar network are functional (Supplementary Fig. 1). The vehicle also uses a smaller piston to create incremental changes in vehicle volume while fully submerged to produce the small changes in density necessary for the vehicle to regulate its depth. The total change in volume that is possible with the small piston is ±0.4% with 12,000 increments over the entire piston range. A geared motor whose shaft is equipped with an optical encoder rotates a threaded rod that controls the small piston. When the piston reaches the end of its translation, the threaded rod then pushes the cylinders apart (Supplementary Fig. 1). In a range of density stratifications off San Diego, USA, the combined system was found to provide adequate control of depth in the upper 50 m of the water column while also providing enough buoyancy at the surface to acquire satellite links.

### Vehicle depth-control algorithm

The M-AUE moves vertically solely via buoyancy control; thus control of vehicle density—and hence position in the water column—is critical. To use the swarm of M-AUEs to measure the horizontal strain of the internal wave field, simple vertical profiling was augmented with the more stringent requirement of depth holding.

**Figure 3:**
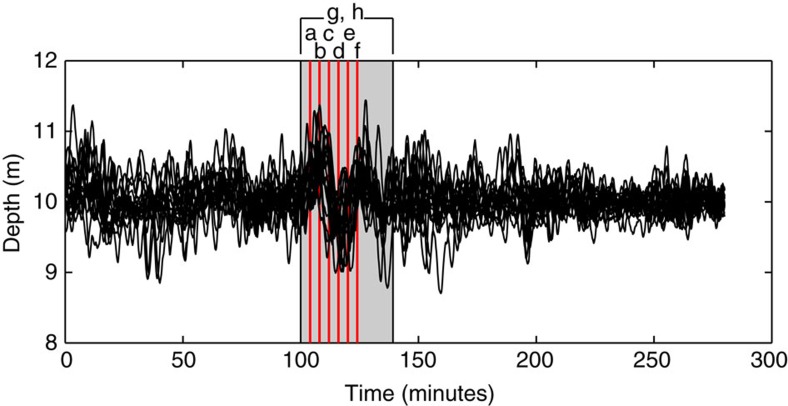
Depth-holding ability of the 16 M-AUEs for the 5-hour at-sea experiment. Once at depth, the M-AUEs maintained their target of 10 m with an s.e. of ± 1.02 m using small motions of the piston to counteract vertical currents and density changes caused by internal waves. Black lines show depths of individual M-AUEs. Red vertical lines show the times of panels **a**–**f** in figure 5, and gray shaded area shows the time period of panels **g** and **h** in figure 5.

Maintaining a given depth requires an algorithm to change the vehicle's buoyancy when ambient currents or changes in density move it vertically. We use the PID control algorithm^23^, which has three adjustable coefficients: the proportional gain, the derivative gain, and the integral gain. These are used to actuate the piston to minimize the difference between the desired depth and the actual depth, with the goal of ultimately converging to the target depth. Although it would be optimal to fix the vehicle's density to achieve a desired density surface, in practice, this is nearly impossible for a compressible vehicle. To accommodate vehicle compressibility, a two-state PID controller was developed: one set of control gains was used at the surface, transitioning to another set when a given target set of conditions was met at depth. In subsequent sea trials, the vehicles were programmed to hold 10 m depth in 40 m of water off San Diego, CA, USA. Over 5 h, the vehicles remained at 10±1.02 m, even during the passage of significant high-frequency nonlinear internal waves (Fig. 3).

### Internal wave measurements by the M-AUE swarm

**Figure 4:**
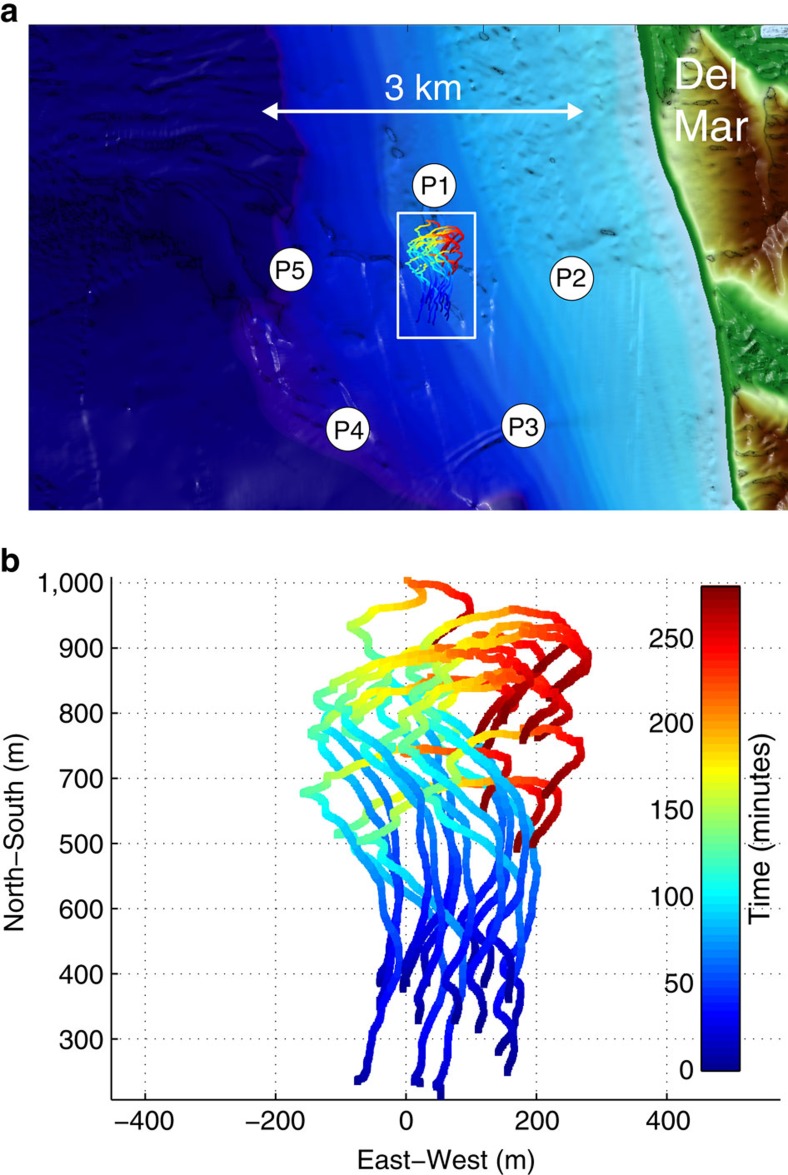
An overview of the field site off Torrey Pines in San Diego. (**a**) The locations of the 5 pingers P1–P5 are shown relative to the local bathymetry. The white box contains a plan view of the measured trajectories of the 16 M-AUEs during the 5-hour deployment. (**b**) The trajectories of the 16 M-AUE once they had reached the target depth of 10 m. Each trajectory is colored according to the time since deployment.

Upon completion of vehicle construction, tank tests and initial sea tests, an experiment was conducted 3 km west of Torrey Pines beach near San Diego, CA, USA over several days in late September–early October 2013. To test accumulation of depth-holding plankton in internal waves, 16 M-AUE vehicles were deployed inside a 3-km diameter pentagonal array of moored surface-float pingers and programmed to maintain depth at 10 m for 5 h (Fig. 4). 3D locations were obtained every 12 s with an estimated s.d. of ±1 m horizontally and <1 cm vertically. An animation of the estimated vehicle positions over the course of the experiment is shown in Supplementary Movie 3. A moored wirewalker^24^ equipped with a CTD provided environmental context through vertical profiles every 7 min.

**Figure 5:**
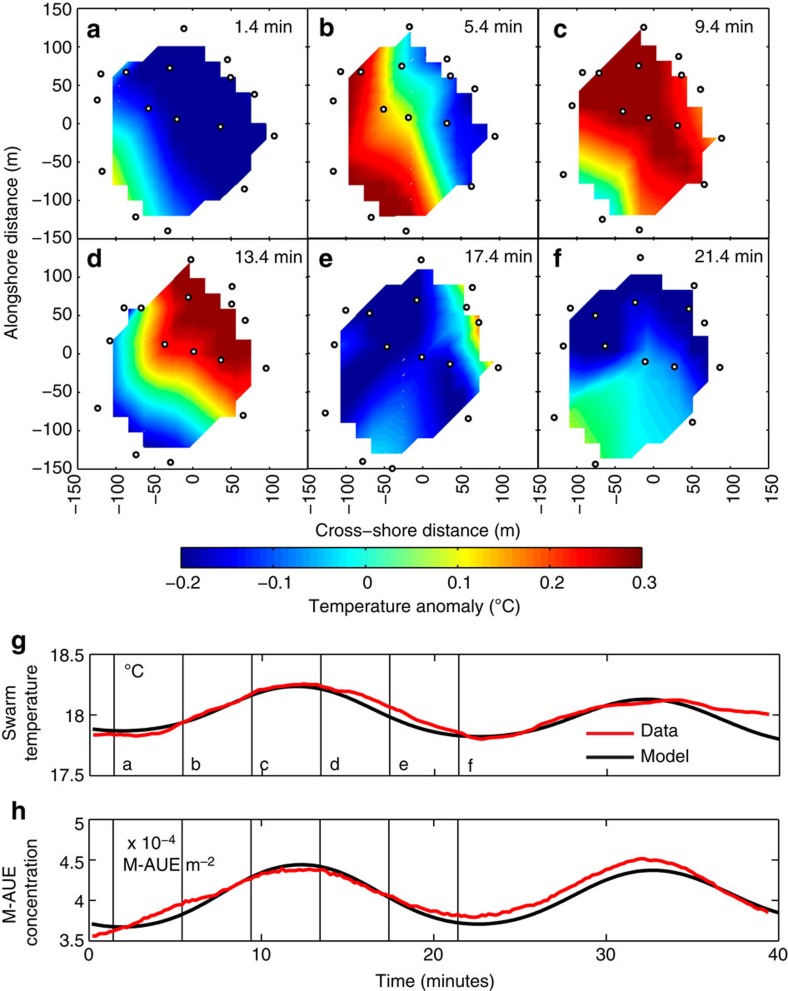
M-AUE swarm showing accumulation in internal wave troughs. (**a**–**f**) Alongshore/cross-shore maps of the temperature anomaly within the M-AUE swarm. Black circles are M-AUE locations in a coordinate system centred at the centre of mass of the swarm. Warm colours indicate internal wave troughs, cold colours the crests. (**g**,**h**) Section of the 5-h time series of average temperature in the M-AUE swarm and the M-AUE concentration (numbers m^−2^), showing changes owing to internal waves propagating through the depth-holding swarm. Red line denotes the data and black line the model (see text for details). Vertical black lines show times corresponding to the six upper panels. Accumulation of M-AUEs over the warm waters of the wave trough supports the model predictions.

To identify high-frequency internal waves, temperature anomalies were calculated as residuals from the 5-h quadratic loess-smoothed temperature records from the individual M-AUEs using a window of 15% of the data. Spatial contours of the temperature anomalies clearly show high-frequency internal waves propagating through the M-AUE swarm (Fig. 5, Supplementary Movie 4).

Theoretical models^13^,^14^,^16^,^17^,^18^ predict that depth-holding objects in the upper water column should accumulate over the troughs of internal waves (that is, in warm water) and disperse over the wave crests (cold water). Changes in concentration of the M-AUEs were calculated from changes in the area of the swarm: smaller areas indicate higher concentrations (Fig. 5). As predicted by theory, the swarm concentrated over wave troughs and dispersed over wave crests. The changes in M-AUE concentration of ∼30% during the 40-min period shown in Fig. 5 were consistent with linear theory^14^; however, the up to 2 × changes in M-AUE concentration over the course of the ∼5-h deployment are indicative of nonlinear high-frequency internal wave activity.

The depth-holding M-AUE swarm acted as a 2D sensing array, enabling accurate detection and quantification of the underlying flows. In particular, the swarm can resolve the wave direction, phase speed, amplitude or the presence of multiple waves with different properties. The wavelength, frequency and phase speed of the dominant (onshore-propagating) waves were diagnosed from cross-correlations of temperature fluctuations in time and space among the M-AUEs (Supplementary Fig. 2). A model of depth-holding objects in a linear internal wave in a linear density gradient^25^ parameterized with these data gave excellent agreement with the changes observed in the M-AUE swarm (Fig. 5). The simple linear theory explained 92% of the observed temperature variance and 88% of the variance of the M-AUE concentration during the 40-min period shown in Fig. 5.

## Discussion

Multi-dimensional sampling by swarms of subsurface vehicles in conjunction with other observational tools such as profiling moorings and even aerial observations will generate data of unprecedented spatial coverage and spatio-temporal resolution, yielding novel insights into physical and biological dynamics at submesoscales in the ocean. Through a 5-h deployment of a swarm of M-AUEs with 3D underwater localization, we have provided a unique, high-resolution realization of individual internal waves *in situ*.

The M-AUEs, programmed to depth hold at 10 m, accumulated over the troughs of internal waves and dispersed over the crests, consistent with theoretical predictions (Fig. 5). Fundamental biological processes in the ocean such as mating, predation and infection depend on the encounter rates among organisms, which typically scale as the product of the concentrations of the organisms involved: male/female, prey/predator, host/parasite, and so on. Thus small changes in concentration of the two types can drive large changes in encounter rates. Here the M-AUEs, acting as plankton mimics, spontaneously formed predictable accumulations without any knowledge of the presence or behaviour of neighbouring vehicles. Peak patch concentrations persisted for 10s of minutes, supporting the hypothesis of internal-wave-enhanced encounter rates among swimming plankton over biologically relevant timescales. The M-AUE swarm has thus enabled the quantification of an ecologically and evolutionarily important physical–biological interaction mechanism in the open ocean.

Though the M-AUE swarm was used here to test a specific hypothesis, the swarm approach and M-AUE technology has great potential to yield significant insights into a wide range of oceanographic problems. The M-AUE drifters are particularly well suited to elucidating subsurface transport pathways, as they are small, self-contained and unencumbered by any surface tether or drogue. M-AUEs are ideal for quantifying larval connectivity of marine protected areas, drift of sperm, eggs and larvae from spawning aggregations, transport of harmful algal blooms or movements of subsurface oil spills, for example. Using large numbers of vehicles will accommodate—and help to quantify—the variance inherent in such flows.

The ability to program the M-AUEs with vertical behaviours, and their small size, makes them excellent mimics for exploring physical–biological interactions of plankton in the ocean. Swarms of isopycnal-following M-AUEs will give unique views into the physical dynamics driving nutrient fluxes at fronts and eddies or the transport pathways of river plumes in the ocean.

Many coastal benthic species have planktonic larval forms that must return to suitable adult habitat to metamorphose. How these larvae cross the shelf is largely unknown. Though little transport is expected from linear internal waves, the large, nonlinear waves often observed in our study region have been hypothesized to be a significant mechanism for transporting invertebrate larvae across the shelf to their adult habitat^26^,^27^. However, the observation that the internal waves passed through the M-AUE swarm showed clearly that the M-AUEs were not transported with the internal waves: the ∼300-m cross-shelf transport that occurred over 5 h was largely driven by the tide.

The M-AUE hydrophone used for subsurface localization also provides a drifting record of the ambient acoustic environment. Ambient sounds have been proposed as a stimulus for larvae to find hospitable underwater habitats^28^; M-AUE swarms could provide unique measurements of the acoustic signals perceived by the drifting larvae.

One particularly attractive aspect of swarms of vehicles is that they form distributed sensing systems. This large (relative to single-point measurements) array of sensors can be used to characterize the underwater acoustic environment or track ships, whales and even seismic events based on passive localization of ambient sounds propagating through the swarm.

The field deployment revealed several limitations of the current vehicle and system design. Vehicle buoyancy is controlled by a relatively slow-moving piston; the slow response limits the vehicle's ability to accommodate rapid vertical water motions, and the motor noise interferes with acoustic reception of the navigation pings. Vehicle tracking would be improved with a faster gear relay, decreasing the amount of time the motor is running. However, this would decrease the accuracy of the depth regulation. A second limitation is that the M-AUEs must be recovered to download their data, which takes 3–5 h. A more advanced system could relay the data via surface Wi-Fi to a surface ship or via satellite to a shore station, with two-way communication allowing reprogramming of the M-AUEs for adaptive sampling. We also envision vehicles computing their own position; with surface float locations and transmit times coded into their acoustic transmissions, an algorithm on the M-AUE could then calculate its location. The main limitation to this is clock drift: drift and synchronization must be accurately known to calculate the absolute time delay between the transmit time of the surface pinger and received waveform by the subsurface vehicle. The use of more modern technologies will obviate this issue as the latest generations of clocks have a drift of one part in a billion, a thousand times more accurate than those currently on the M-AUEs. This will permit submillisecond synchronization facilitating much longer deployments and allow the one-way travel time to be computed by the M-AUE while submerged, given the known transmit time of the ping from the surface floats.

Larger excursions—longer deployments—of the M-AUE swarm will require a larger array of surface acoustic transmitting floats to track the drifting vehicles. Presently, the M-AUEs can detect pings from the surface floats with a reasonable signal-to-noise ratio at a range of 5–6 km. This distance constrains the size of the array containing the M-AUE swarm. As an alternative to a moored array, we are experimenting with a mobile navigation net in which the surface float pingers are attached to GPS-tracked drogued drifters. This surface array drifting around the swarm would allow tracking of the M-AUE swarm over much longer times and distances. Another potential alternative for extended subsurface localization is the use of ambient sound, in which relative position could be estimated from inter-M-AUE correlations with external sound sources such as a GPS-localized boat that is driven around the swarm. These schemes take advantage of passive vehicle localization: because the vehicles do not sonically interfere with each other (except for their motor noise), swarms of hundreds or even thousands of such vehicles could be deployed with concomitant gains in sampling at ever-finer scales and/or over larger distances.

Here we have described the M-AUE: an autonomous, buoyancy-controlled drifter with continuous 3D underwater localization. These small, relatively inexpensive vehicles are ideal for deployments as a swarm for exploring physical and biological dynamics on scales of metres to kilometres and minutes to days. Field deployments have demonstrated their utility as both a large-scale sensing array for high-frequency internal waves and as plankton mimics with preprogrammed vertical swimming behaviours. Use of these M-AUE swarms will provide unique and powerful new insights into a wide range of otherwise intractable physical and biological problems in the ocean.

## Methods

### The M-AUE system components

The M-AUE is controlled by a PIC32 microcontroller (PIC32MX695F512L, Microchip) with a clock rate of 80 MHz and 128 kB RAM. Sensor data, motor events and communications are handled through interrupt routines, as the controller employs no operating system. A 32 GB SD card (SanDisk) stores all sensor, hydrophone and system data in a packetized file format. System local time, GPS time and GPS position, recorded when the unit is on the surface, are saved in a separate file.

A custom linear actuator made from a Delrin piston, stainless steel lead screw, worm gear and two Firgelli gear motors is used to control buoyancy and also extend the housing when at the surface. Significantly, the motor mounts were 3D printed to yield precise positioning of the motors relative to the worm gear, resulting in a very compact linear actuator with a stall force of 200 lbs.

Hydrophone data provided by a HTI 96-Min with −161 dB receive sensitivity, (High Tech Inc., Long Beach, Miss) are sampled with a resolution of 16 bits and a rate of 65536 Hz. A pressure sensor (Measurement Specialties, 86 series), temperature sensor (Minco S614PDX12T), 3-axis accelerometer (Memsic MXR9500GZ) and Compass (Honeywell, HMC6343) are sampled at 16 Hz. In addition to these sensors, internal temperature, battery charge, battery voltage, system status, control algorithm state and settings are also sampled at 16 Hz.

The system is powered by a battery pack made from 6 Tenergy 1.2 V, 5000, mA hour NiMH cells in a series configuration. The battery pack is fused with a 2 A fuse and is recharged at an optimal rate of 600 mA.

The system has a dedicated board for GPS, RF, Globalstar, MiWi and light-emitting diode communications. A u-blox6 GPS receiver is used with a Sarantel Helical GPS antenna (GeoHelix SL1300) to yield good localization near the water surface. For additional surface localization, a Semtech SX1230 RF transmitter is programmed to send GPS coordinates from the unit over an RF link at 434 MHz with a usable range of 500–1,000 m. An STX2 Globalstar transmitter sends GPS coordinates over the Globalstar satellite network. These coordinates are relayed to cell phones via the Geoforce system. Unit positions are also available on the Geoforce website.

Average vehicle power consumption is quite stable around 0.6 W during both recovery and deployment modes. While the motor, Globalstar and RF transmissions use significantly more power, they are brief and intermittent and the power consumption of the PIC32 (always in active mode) is by far the dominant power drain on the system.

### The surface pinger components

Each surface pinger, measuring 160 × 13 cm^2^, consists of a microcontroller (PIC32MX695F512L, Microchip), GPS receiver (Ublox6) with helical antenna (GeoHelix SL1300, Sarantel), Acoustic transducer (Benthos 013701) with power amplifier (Benthos C-855-130) and 12 V, 20 A hour battery pack (BatterySpace 7626). Pinger battery life with continuous operation is approximately 1 week. The components are held together using a long aluminum frame and are housed in a clear acrylic tube with the battery pack at the bottom to provide a large separation between the centre of mass and centre of buoyancy and, hence, promote stability.

The microcontroller receives a precisely timed interrupt from the GPS receiver each second when there is a valid fix and GPS time lock. The accuracy of the time pulse output of the receiver is very high (∼10 ns). The PIC32 samples this pulse with a resolution of 1 clock cycle (12.5 ns). Additional latencies in the waveform generation and transmission yield an effective accuracy of roughly 1 μs.

A frequency modulated square wave is generated on the PIC32 and used to drive a class B amplifier (Benthos C-855-130) to produce an approximation to a linear frequency modulated chirp of the form:


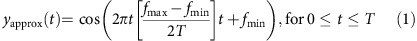


where *f*_min_=8 kHz, *f*_max_=15 kHz and *T*=10 ms. This waveform is transmitted once every 12 s with a pinger-specific time offset to ensure each ping is separated in time and identifiable. During operation, the pinger logs GPS position, GPS time (in Time Of Week format with millisecond resolution), transmit signal type, pinger id number and a binary indicator of whether or not a ping was sent at the given GPS time of week.

### The depth–control algorithm

The control algorithm used with the M-AUE is based on a PID regulator. As the aim is to go to some target depth, the PID controller computes the error *e(t)* over time as the difference between the present depth *d(t)* and the target depth *d*_*T*_:


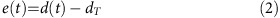


The control signal *c(t)* driving the piston position depends directly on this error:


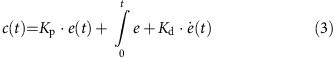


where *c(t)* controls the displacement of the piston, a value between its minimum and maximum value. We note that *c(t)* is in counts as this reflects the linear actuator's absolute position as encoded by the optical encoder. In the equation, *K*_p_ is the proportional gain, *K*_i_ is the integral action, *K*_d_ is the derivative action, and *ė(t)* is the derivative of the error. In practice, tuning the parameters required a reduction in the integral gain; however, this led to a persistent overshoot and a bias in the depth converged to. Thus another term was added that we call the neutral position: *N*_p_. *N*_p_ is computed similarly to the integral action term, but the error is added to the neutral position only if the error derivative is small enough. This minimizes the overshoot owing to the high momentum of the M-AUE, leaving the final obtained depth unbiased. Incorporating *N*_p_ as the last term, the control is now:


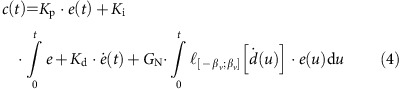


Here *G*_*N*_ is a neutral adjust gain and *ℓ*_[−*βv*;*βv*]_ is a dead-band constant that turns this term on when the velocity is within the interval [−*β*_*v*_;*β*_*v*_] and off when the velocity is outside of the interval.

Though the algorithm worked well in a 10-m test tank to hold depth at 5 m, at sea the vehicles oscillated substantially around the 16-m target depth. Because extensive field trials exploring a variety of parameters were not possible, a computer model of the vehicle's behaviour was created. The model included static factors such as the vehicle's mass, water density, vehicle radius, volume of air at the surface, an added mass drag coefficient, a surface tension effect and vehicle elasticity. Testing a range of tuning parameters for the PID model with added velocity limitations showed that, because of vehicle compressibility, the controller was not capable of providing depth holding with a small residual.

Based on the modelling results, the PID controller was modified to create a two-state model, state 1 being a transitory initial state, and state 2 a stationary state. The second state has a new set of parameters for proportional gain, a zero integral gain to eliminate the historical effects of the previous transitory behaviour, an identical derivative gain and a new neutral adjust gain (Supplementary Table 1). To reset the piston's neutral position to an optimal value after transitioning, we calculate the average piston position within a transition-time window over time interval of duration *τ*, 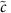
(*t*_*1*_:*t*_*2*_). The final control output is computed as:


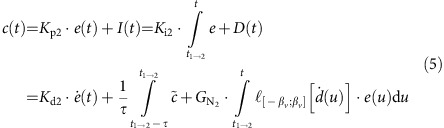


The algorithm also implemented a limit on the smallest rate of change of the piston in order to conserve battery power. This was adaptively changed from a state 1 value to zero in state 2, permitting very small adjustments.

### System deployment

Supplementary Movie 1 demonstrates the deployment of the surface pingers. Supplementary Movie 2 demonstrates the deployment of the subsurface floats.

### Data analysis

The area of the 16 vehicle swarm was calculated every 12 s during the 5-h deployment from the M-AUE locations using the *convhull* command in Matlab. The phase speeds of internal waves were estimated from cross-correlations of the temperature signals of the M-AUE farthest offshore and all other M-AUEs (Supplementary Fig. 2). The slope of the linear regression of the time lag giving the maximum cross-correlation versus the separation distance between the M-AUEs gave a phase speed of 0.29 m s^−1^. The wave amplitude was estimated from the measured temperature variations at a given M-AUE and the vertical temperature gradient was measured by the wirewalker CTD. Internal wave temperature anomalies were calculated using the *smooth* command in Matlab as the residual of loess-smoothed temperature records using 15% of the data for each M-AUE. The wave period was estimated from the time between peaks or troughs of the temperature anomalies at each M-AUE and the horizontal wavenumber from the distance between wave crests or troughs as they propagated through the swarm.

### Internal wave model

The linearly stratified linear internal wave model of Thorpe^25^ (his example 3.3a) was chosen for comparison to the M-AUE data. The wave parameters were taken from the M-AUE swarm, assuming all density variations were due to temperature. Virtual M-AUEs with perfect depth holding were moved by the flows calculated from the stream function


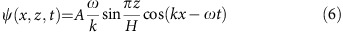


where *A* is the wave's amplitude (2.67 m), *ω* the frequency (0.0056, s^−1^), *k* the horizontal wavenumber (0.0193, m^−1^) and *H* the water depth (40 m).

### Data availability

Data are available from the authors upon request.

## Additional information

**How to cite this article**: Jaffe, J. S. *et al*. A Swarm of autonomous miniature underwater robot drifters for exploring submesoscale ocean dynamics. *Nat. Commun.* **8**, 14189 doi: 10.1038/ncomms14189 (2017).

**Publisher's note**: Springer Nature remains neutral with regard to jurisdictional claims in published maps and institutional affiliations.

## Supplementary Material

Supplementary Information Supplementary Figures and Supplementary Table

Supplementary Movie 1 A video showing the deployment of the pingers at sea.

Supplementary Movie 2 A video of the deployment of the M-AUE drifters at sea.

Supplementary Movie 3 A video that illustrates the trajectories of the M-AUE vehicles over the 5 hour experiment that was performed offshore of Torrey Pines, San Diego, on Oct 1, 2013.

Supplementary Movie 4 An animation of the high-frequency internal wave temperature anomalies moving through the M-AUE swarm during a deployment offshore of Torrey Pines, San Diego, on Oct 1, 2013. The animation shows a plan view following the center of mass of the swarm. The numbers show the locations of the individual M-AUEs.
