# Supplementary material for: A swarm of autonomous miniature underwater robot drifters for exploring submesoscale ocean dynamics
Source: Nat Commun. 2017 Jan 24;8:14189. doi: 10.1038/ncomms14189 (PMC5286117; doi:10.1038/ncomms14189)
Supplement: Supplementary Information — Supplementary Figures and Supplementary Table [file ncomms14189-s1.pdf]

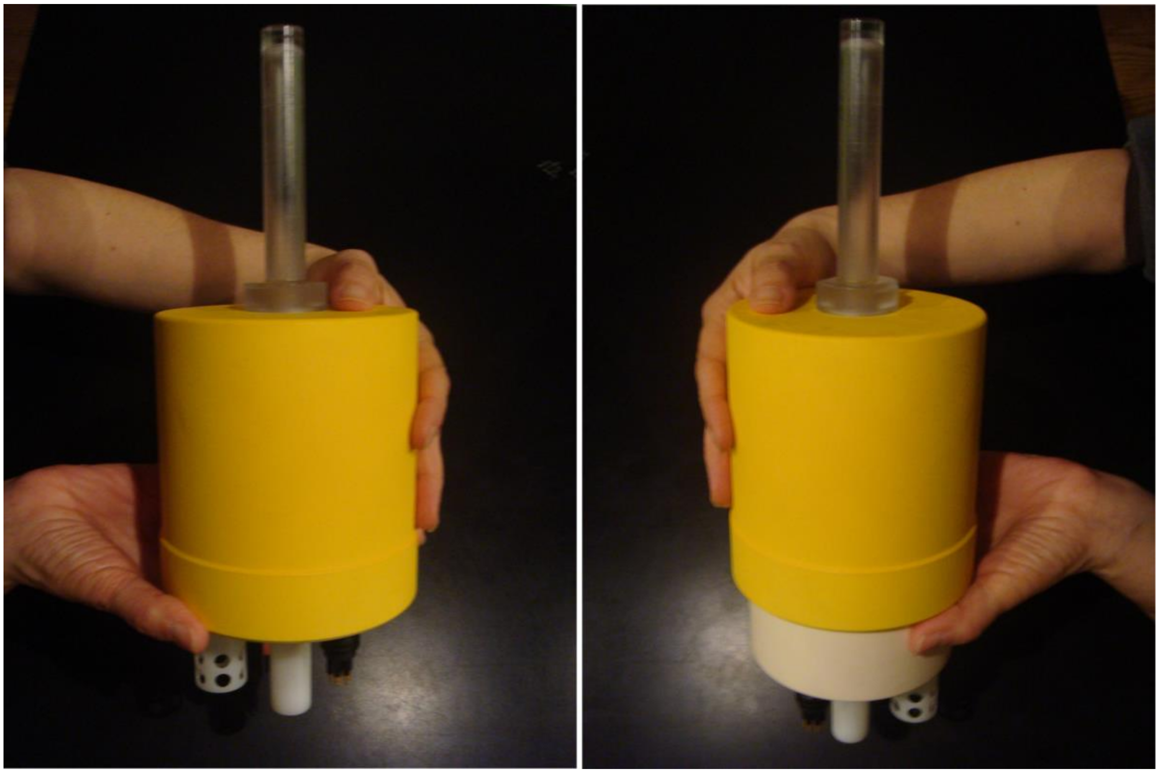

Supplementary Figure 1. **Expansion of the outer M-AUE housing.** The image shows the retracted (left) and expanded (right) housing with the inner sleeve protruding from the bottom of the vehicle.

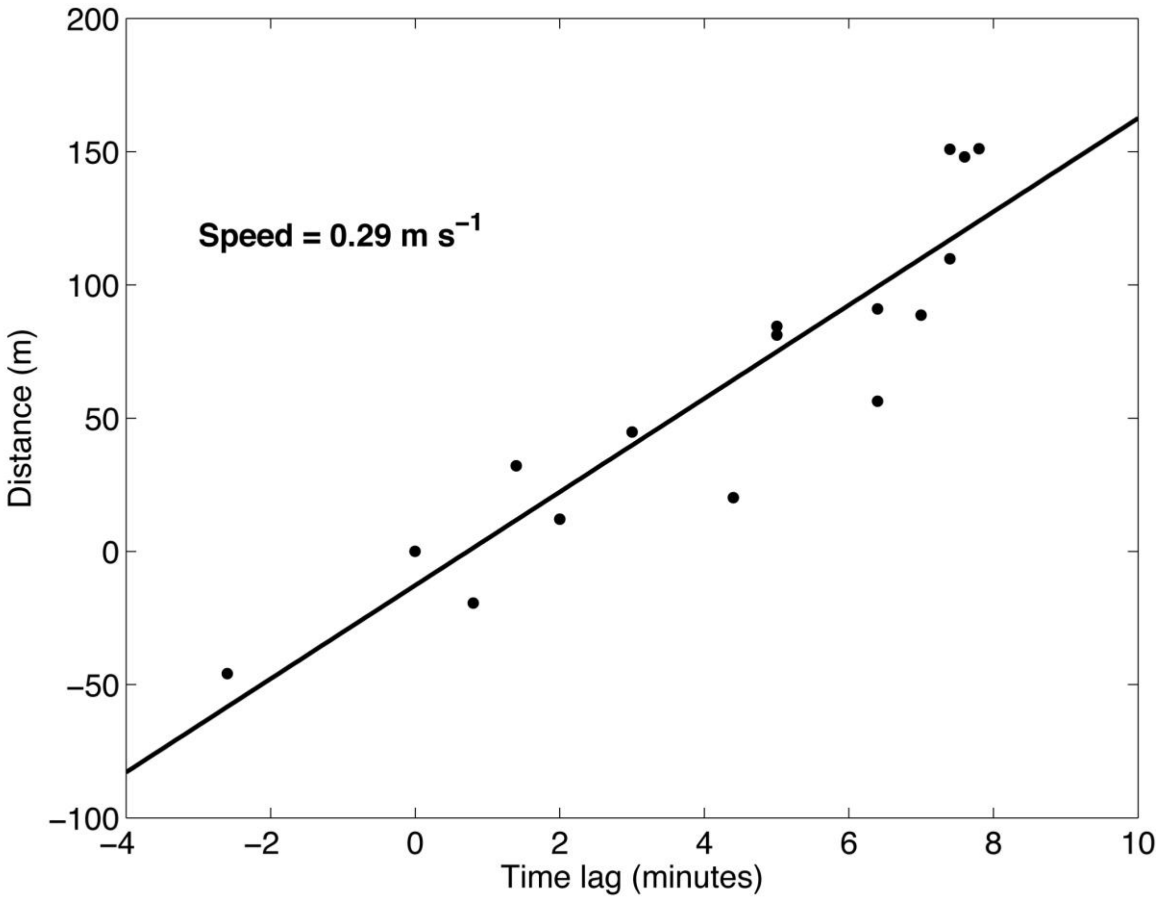

Supplementary Figure 2. Calculation of the phase speed of the internal wave. The phase speed of the wave is given by the slope of the regression line of the time lag giving the maximum cross-correlation of the temperature records of a given M-AUE relative to the M-AUE farthest offshore, and the separation distances of the pairs of M-AUEs.

Supplementary Table 1. The two-state PID control parameters. State 1 parameters get the M-AUE to depth, transitioning to the state 2 parameters to keep it at depth.

	State 1	State 2
Proportional gain	$K_p = 500$	$K_{p_2} = 250$
Integral gain	$K_i = 15$	$K_{i_2} = 0$
Derivative gain	$K_d = 9200$	$K_{d_2} = 9200$
Neutral adjust gain	$G_N = 5$	$G_{N_2} = 2$
